# Rapid Label-Free Analysis of Brain Tumor Biopsies by Near Infrared Raman and Fluorescence Spectroscopy—A Study of 209 Patients

**DOI:** 10.3389/fonc.2019.01165

**Published:** 2019-11-05

**Authors:** Roberta Galli, Matthias Meinhardt, Edmund Koch, Gabriele Schackert, Gerald Steiner, Matthias Kirsch, Ortrud Uckermann

**Affiliations:** ^1^Clinical Sensoring and Monitoring, Anesthesiology and Intensive Care Medicine, Faculty of Medicine Carl Gustav Carus, Technische Universität Dresden, Dresden, Germany; ^2^Neuropathology, Institute of Pathology, University Hospital Carl Gustav Carus, Technische Universität Dresden, Dresden, Germany; ^3^Neurosurgery, University Hospital Carl Gustav Carus, Technische Universität Dresden, Dresden, Germany

**Keywords:** glioma, meningioma, brain metastases, schwannoma, biopsies, Raman spectroscopy

## Abstract

In brain surgery, novel technologies are continuously developed to achieve better tumor delineation and maximize the extent of resection. Raman spectroscopy is an optical method that enables to retrieve a molecular signature of tissue biochemical composition in order to identify tumor and normal tissue. Here, the translation of Raman spectroscopy to the surgical practice for discerning a variety of different tumor entities from non-neoplastic brain parenchyma was investigated. Fresh unprocessed biopsies obtained from brain tumor surgery were analyzed over 1.5 years including all patients that gave consent. Measurements were performed with a Raman microscope by medical personnel as routine activity. The Raman and fluorescence signals of the acquired spectra were analyzed by principal component analysis, followed by supervised classification to discriminate non-tumor tissue vs. tumor and distinguish tumor entities. Histopathology of the measured biopsies was performed as reference. Classification led to the correct recognition of all non-neoplastic biopsies (7/7) and of 97% of the investigated tumor biopsies (195/202). For instance, GBM was recognized as tumor with a correct rate of 94% if primary, and of 100% if recurrent. Astrocytoma and oligodendroglioma were recognized as tumor with correct rates of 86 and 90%, respectively. All brain metastases, meningioma and schwannoma were correctly recognized as tumor and distinguished from non-neoplastic brain tissue. Furthermore, metastases were discerned from glioma with correct rate of 90%. Oligodendroglioma and astrocytoma *IDH1*-mutant, which differ in the presence of 1p/19q codeletion, were discerned with a correct rate of 81%. These results demonstrate the feasibility of rapid brain tumors recognition and extraction of diagnostic information by Raman spectroscopy, using a protocol that can be easily included in the routine surgical workflow.

## Introduction

During the last decades, advances in imaging, functional mapping, neuronavigation as well as fluorescence-based technologies for identification of malignant tissue have increased the ability of neurosurgeons to optimize tumor removal and preserve normal brain (1). However, tissue biopsies are still collected inside the neoplastic lesion to enable an intraoperative consultation that provides a preliminary diagnosis (2). Intraoperative neurosurgical histopathology relies on evaluation of rapid tissue preparations, is time consuming and extends the duration of surgery. Furthermore, the soft nature of nervous tissue leads to low-quality frozen sections (3).

Optical molecular imaging techniques emerged in the recent years as innovative methods for retrieving diagnostic information and provide real-time histopathologic images of tumors (4). Among those techniques, Raman spectroscopy has attracted increasing attention in oncology as non-invasive and label-free method, with the aim to provide new tools for the detection of malignant and pre-malignant lesions in a variety of cancer entities (5). Raman spectroscopy relies on the excitation of inelastic light scattering processes inside the molecules that build the tissue. The spectroscopic analysis of the inelastically backscattered light, generated upon sample irradiation with a low-power laser beam, provides information about the overall biochemical composition. The Raman spectrum is composed of bands whose position is correlated with the characteristics of molecular bonds (i.e., functional groups). Raman band intensity is correlated with the concentration of those groups, thereby providing the possibility to probe the biochemistry of cancerous tissue without markers and to perform molecular pathology (6–8).

In neuro-oncology, Raman spectroscopy proved to be suited for discerning brain tumors from normal brain parenchyma based on several changes of neoplastic tissue composition, which include decreased lipid content, altered protein profile, and increased nucleic acid levels (9–15). Based on the specific combination of these alterations, Raman spectroscopy enables to retrieve information about tumor type (16), malignancy grade (17–19), molecular profile (20), and recurrency (21), as well as to recognize necrosis (22–24) and to detect infiltrative regions (25).

The aforementioned studies mainly used experimental brain tumor models, or fixed samples and cryosections of human tissue. Especially when using fresh samples, the number of patients included in each study was small and, generally, each study focused on one or a few types of brain tumor. These studies provided strong evidence of the capabilities of Raman spectroscopy for brain tumor delineation and identification, but were still configured more as biochemical rather than clinical research. Recently, it was shown that Raman spectroscopy enables intraoperative recognition of glioma *in situ* (26), which highlights the impact that such a technology may have in neurosurgery during the next decades and motivates to move further in the direction of clinical translation.

Despite the huge advances observed in the last years, the evidence that most types of brain tumors can be distinguished from normal tissue without pre-existing information about tumor type was not provided yet. A translation of the method into the clinical practice and its integration in the routine surgical workflow are lacking as well.

Therefore, we performed Raman spectroscopic measurements of fresh, unprocessed intraoperative biopsies with the double aim to discriminate non-tumor tissue vs. neoplastic tissue of a variety of tumor entities, and to demonstrate the feasibility of measurements during routine surgery. The measurements were thus performed directly by medical personnel for a time period required to obtain a statistically significant amount of data. All brain tumor types were included in the study and biopsies obtained from drug-resistant epilepsy surgery were used as non-neoplastic control. For tumor recognition, we exploited a combination of the spectroscopic information contained in both near infrared fluorescence and Raman signals. This approach differentiates the present study from other research, where the fluorescence was always considered a disturbance and eliminated from the spectra with a baseline procedure.

## Materials and Methods

### Ethics Statement and Biopsy Collection

The study was approved by the ethics committee at the Dresden University Hospital (EK 323122008). The biopsies were obtained from brain surgeries where patients gave informed consent.

### Raman Spectroscopy

The spectrometer is a RamanRxn (Kaiser Optical Systems Inc., Ann Arbor, USA) coupled to a light microscope (DM2500 P, Leica Microsystems GmbH, Wetzlar, Germany). The excitation of Raman scattering was performed with a diode laser (Invictus 785-nm NIR, Kaiser Optical Systems Inc., Ann Arbor, USA) emitting at a wavelength of 785 nm with a maximum power of 400 mW. The laser excitation was propagated to the microscope with a multimodal optical fiber with core diameter 100 μm and focused on the samples by means of a 10×/0.25 microscope objective (N Plan, Leica Microsystems GmbH, Wetzlar, Germany), leading to a focal spot of about 80 μm. The laser power measured at the sample position was set to 200 mW. The laser source is safety class 3B and was handled in accordance with national safety regulations. An enclosure of the system equipped with laser interlock guarantees protection from reflected or scattered laser light during acquisition as well as rejection of ambient light. The scattered light was collected in reflection configuration and sent to the spectrograph by using a multimodal optical fiber with core diameter 62.5 μm. The acquired spectral range was from 350 to 3,250 cm^−1^ (relative to excitation) and the spectral resolution was 4 cm^−1^. For each point measurement, 20 Raman spectra were acquired with 2 s integration time and averaged. Two to ten measurements (typically five) were performed on each biopsy.

### Histology

After Raman spectroscopy, the biopsies were fixed in 4% formalin in phosphate buffered saline and cryoprotected in rising concentrations of sucrose (10%, 30% for 24 h, respectively). Embedding in tissue freezing medium was followed by storage at −80°C until preparation of 10 μm thick cryosections, which were subjected to hematoxylin and eosin (H&E) staining. The sections were washed in aqua dest and incubated in Meyer's hematoxylin/hemalum for 3 min. After washing in aqua dest, the tissue was briefly destained in HCl-ethanol. Washing using tap water for 5 min was followed by 3 min staining in eosin (1% eosin G in 80% ethanol). The sections were dehydrated in rising ethanol concentrations, cleared in xylene and coverslipped using DePex.

### Spectroscopic Data Analysis

Spectroscopic data were processed and analyzed in Matlab (MathWorks Inc., Natick, MA, USA). Statistical analyses were performed with Prism 6.0 (Graph Pad Software Inc., La Jolla, CA, USA).

The fluorescence signal was retrieved with a baseline procedure. A variable baseline was calculated for each raw spectrum by applying the function “msbackadj” of the Bioinformatics Toolbox. The baseline was estimated within multiple spectral windows of width 200 cm^−1^, shifted with 200 cm^−1^ steps and linear interpolation. The intensity of the fluorescence was obtained as area under the baseline curves. The fluorescence provides the largest contribution to the acquired signal intensity, accounting for 92.5% of the total average intensity in the acquired spectral range (lowest contribution for meningioma: 88.4%; highest contribution for non-tumor tissue: 96.5%).

The Raman signal was obtained by subtracting the baseline from the raw spectra. Afterwards, the spectral range was reduced by excluding all regions without Raman signals or with ambient light artifacts. The spectral ranges used for subsequent analysis were: 478–1,707 cm^−1^, 2,578–2,877 cm^−1^, 2,890–3,027 cm^−1^. The region between 1,707 and 2,578 cm^−1^ was excluded as it does not contain any Raman band of biological component (silent region). The data in the range 2,877–2,890 cm^−1^ were excluded as some spectra contained ambient light artifacts in this region. Subsequent normalization of the set of spectra was obtained by standardizing the area under the curve to the group median value by using the function “msnorm” of the Bioinformatics Toolbox.

Raman band intensity was calculated as maximum of the band. Two-tailed Mann-Whitney rank test was used for statistics as the data were not found to be normally distributed.

Principal component analysis (PCA) was applied for dimensionality reduction of fluorescence and Raman spectra using in both cases the Matlab function “princomp.” Supervised classification was performed by quadratic discriminant analysis using the PCA scores. The function “classify” of Matlab was used for the purpose. The scores of the spectra of one biopsy were used as test set and classified using the scores of the spectra of all other biopsy as training set. The procedure was cycled on all biopsy. The classification provides a probability of class membership for each spectrum (the so called posterior probability), which was then used to retrieve the mean probability for each biopsy. The tissue was analyzed with respect the probability to be neoplastic, i.e., when probability was >0.5 then assignment to the tumor class was done. For combined classification, a mean value of the posterior probabilities obtained from discriminant analysis of fluorescent and Raman data was calculated for each spectrum and then used to calculate the mean probability for each biopsy.

For classification of non-neoplastic vs. neoplastic tissue, the number of scores to be used for classification was found by analyzing the correct rate of both classes obtained with increasing number of scores (Supporting Figure S1). The optimal number was set corresponding to the (local) maximum of the overall classification rate if the correct rate of non-tumor tissue was above 80%. This was found with 11 PCs for the classification of fluorescence spectra and 14 PCs for the classification of Raman spectra.

For tumor type classification, the maximally discriminant scores were selected based on statistical significance of differences between the two classes. For this analysis, ranking of PCA scores was performed based on the Fisher criterion (27). The Fisher rank was calculated for each score. This parameter is high for features having high mean inter-class separation and small within-class variance, and it was used to determine the scores to be used for classification. In the classification of glioma vs. metastatic biopsies, nine scores comprised between 1 and 31 were used for fluorescence and 17 scores between 1 and 23 for Raman spectra. In the classification of astrocytoma *IDH 1*-mut vs. oligodendroglioma, 12 scores comprised between 1 and 19 were used for fluorescence and eight scores between 1 and 29 for Raman spectra.

## Results

Fresh unprocessed biopsies of 210 patients undergoing brain surgery at the Dresden University Hospital were collected in the operating room (OR) immediately after resection (one biopsy for each patient) and transported to the Raman spectroscopic system inside a tube filled with isotonic NaCl solution. For spectroscopy, the tissue was extracted from the tube and immediately placed under the Raman microscope. One biopsy of brain tumor metastases fell apart in the tube and no tissue for the measurements could be recovered. The remaining 209 biopsy were analyzed. All measurements took place within 30 min from tissue resection. Punctual Raman spectra were recorded by two nurses, which were trained for the purpose, but without supervision of a scientist.

The experiments spanned 18 months and included all surgeries where patients gave consent and the surgeon was able to provide spare tissue samples for research purposes. Therefore, the composition of the experimental group (Table 1) mirrors the epidemiology of brain tumors in Germany and includes glioma, brain metastases, meningioma, schwannoma, as well as a heterogeneous group of less frequent tumors that were grouped as “others.” Necrotic tissue was obtained from resection of recurrent glioblastoma and metastases. Supporting Table S1 reports all patients' diagnosis, which were obtained by routine histopathological analysis on biopsies different from the ones used for this study.

**Table 1:** Analyzed tumor types.

**Tissue type**	**No. of patients**	**No. of spectra**
Non-tumor	7	37
Astrocytoma	14	80
WHO II	2	10
WHO III	12	70
Oligodendroglioma	10	50
WHO II	1	5
WHO III	9	45
Glioblastoma	52	261
Glioblastoma recurrent	21	105
Necrosis	4	25
Metastases	23	119
Adenocarcinoma	17	89
Squamous cell carcinoma	4	20
Melanoma	2	10
Meningioma	53	265
WHO I	36	180
WHO II	17	85
Schwannoma WHO I	8	38
Others	17	90
Total	209	1070

Non-neoplastic tissue was obtained from hippocampi resected during surgical treatment of drug-resistant epilepsy. The tissue biopsies investigated were additionally analyzed by a trained neuropathologist on H&E stained cryosections (Supporting Figure S2). In all biopsies, the presence of neoplastic alterations was excluded. One biopsy originating from a patient affected by ganglioglioma (patient 83) displayed, however, an abnormally increased cell density.

Five spectra were acquired at different positions for each tissue sample. In a few cases, fewer spectra were acquired on very small biopsies. Up to 10 spectra were acquired on very large biopsies, among which were some hippocampi. The number of spectra is indicated in Table 1 as well. The fluorescence and Raman signals contained in the raw spectra were then separated, analyzed to extract biochemical differences and finally exploited for tumor classification.

### Analysis of the Spectroscopic Information

**Figure 1:**
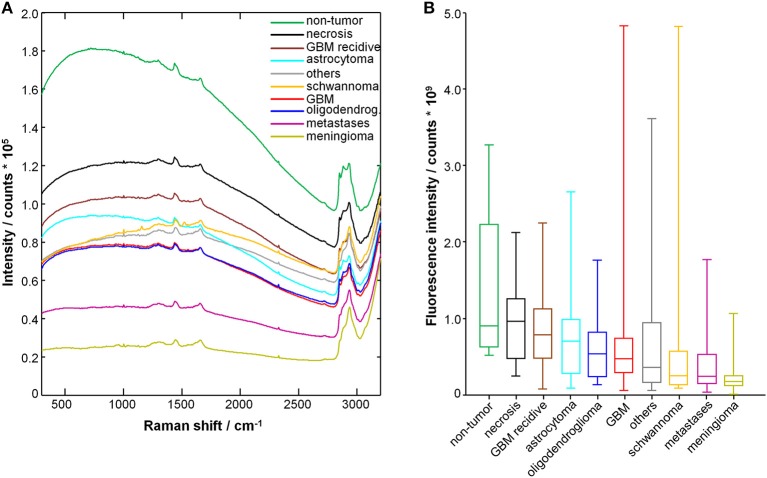
Spectra of tumor and non-tumor tissue biopsies. **(A)** Mean raw spectrum acquired for each tissue type; the Raman bands are superimposed with a large fluorescence signal. **(B)** Fluorescence signal intensity; median, box: 25th−75th percentiles, whiskers: min-max.

Figure 1A shows the average raw spectra of all types of tumors. The Raman signal is often superimposed to strong fluorescence. In fact, the fluorescence provides the largest contribution to the acquired signal intensity. Overall, the different tumor entities display notably different fluorescence signal intensities. Non-tumor tissue displays the highest one. Furthermore, differences in the spectral shape were observed, as it can be seen for schwannoma as best example.

The fluorescence signal intensity was quantified for each spectrum and the results are shown in Figure 1B. The fluorescence signal of non-tumor tissue and of necrosis is significantly higher compared to all glial tumors, metastases, meningioma, schwannoma, and other tumors (*P* < 0.001, two-tailed Mann-Whitney test). The fluorescence signal of non-tumor tissue is also significantly higher compared to recurrent GBM (*P* < 0.01, two-tailed Mann Whitney test). Interestingly, recurrent GBM displays fluorescence signal intensities significantly higher than GBM (*P* < 0.001, two-tailed Mann-Whitney test), but close to necrosis.

**Figure 2:**
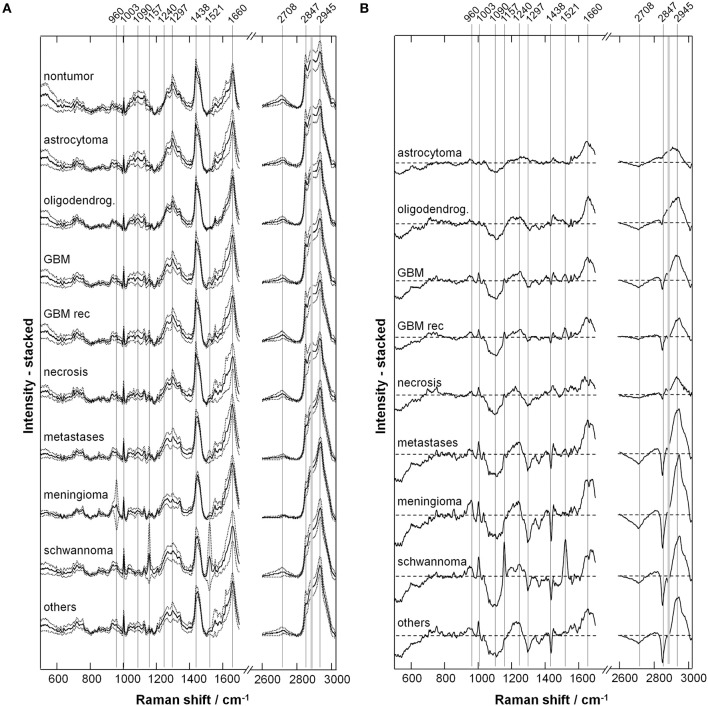
Extracted Raman signals. **(A)** Representative spectrum for each tumor type (mean ± SD). **(B)** Difference spectrum (tumor—non-tumor). The bands indicated by gray lines show variations between neoplastic and non-neoplastic tissue; their assignment is given in Table 2.

Figure 2A shows the representative Raman spectra for each type of tumor, and Figure 2B shows the difference spectra obtained by subtracting the mean spectrum of non-neoplastic tissue from the mean spectra of the different tumor types. The assignment of bands to molecular vibrations (28, 29) is given in Table 2. Non-neoplastic tissue is characterized by intense bands of lipids at 1,090, 1,297, 1,438, 2,708, and 2,847 cm^−1^. These bands progressively decrease in glioma, metastases and meningioma, accounting for the lower lipid content of tumor compared to normal brain tissue. All tumor types display higher bands of proteins at 1,003, 1,240, 1,660, and 2,945 cm^−1^. The overall spectral differences compared to normal tissue are lowest for astrocytoma. The spectra of schwannoma and of GBM (also recurrent) are additionally characterized by two bands at 1,157 and 1,521 cm^−1^, which account for presence of carotenoids. The spectrum of meningioma contains a band at 960 cm^−1^, which indicates presence of calcifications.

**Table 2:** Raman band assignment; ν: stretching, δ: deformation.

**Band position (cm^**−1**^)**	**Vibration**	
960	ν(${\text{PO}}_{4}^{3-}$)	Hydroxyapatite
1003	ν(C-C) ring	Phenylalanine
1090	ν(C-C) skeletal; ν(${\text{PO}}_{2}^{-}$)	Acyl chain lipids; phospholipids
1157	ν(C = C)	Carotenoids
1240	Amide III	Proteins
1297	δ(CH_2_)	Lipids
1438	δ(CH_2_)	Lipids
1521	ν(-C = C-)	Carotenoids
1660	Amide I	Proteins
2708	ν (CH_2_) olefinic	Lipids
2847	ν (CH_2_) aliphatic	Acyl chain lipids
2945	ν (CH_3_)	Proteins

**Figure 3:**
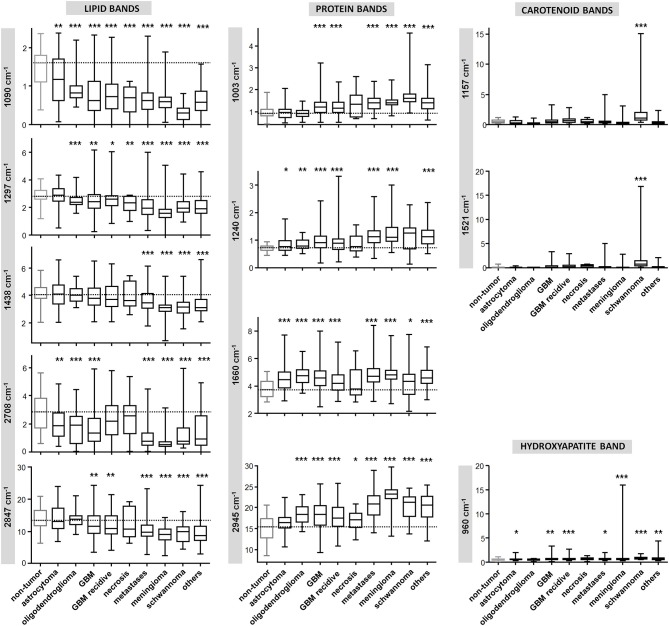
Quantification of Raman band intensity. Quantification of intensity of Raman bands indicated in Figure 2 and assigned in Table 2. The scale of vertical axes is the Raman intensity in arbitrary units. Two-tailed Mann-Whitney test: *P < 0.05, **P < 0.01, ***P < 0.001.

Figure 3 shows the quantification of intensity of the Raman bands discussed above. In agreement with the qualitative changes visualized by Figure 2, most lipid bands are significantly decreased in tumor compared to non-tumor tissue. The largest decrease is observed for high grade entities like GBM and metastases, and for tumors that do not originate from brain cells, like meningioma. The bands at 1,090 and 2,708 cm^−1^ display a significant decrease for all tumor types, including also astrocytoma and oligodendroglioma, while this is not the case for bands at 1,297, 1,438, and 2,847 cm^−1^. The band at 1,090 cm^−1^ has a component from the phosphodioxy vibration of brain phospholipids (30), and is the most sensitive toward neoplastic brain transformation. The bands at 1,297, 1,438, and 2,847 cm^−1^ are produced by C-H vibrations of acyl chain CH_2_ groups, and are less specific toward detection of brain lipids alterations. Protein bands tend to be significantly increased in all tumors. The band at 1,240 cm^−1^ contains contributions of collagen (15), and is thus more intense in the spectra of meningioma, schwannoma, metastases and GBM, which are tumors that more often display a collagenous extracellular matrix and/or hypervascularization. The quantification of carotenoid bands at 1,157 and 1,521 cm^−1^ confirms that these are characteristic for schwannoma; they are present in a subgroup of GBM (both primary and recidive) and metastases as well, but the difference compared to non-tumor tissue is not significant. The band of hydroxyapatite at 960 cm^−1^ is significantly increased is many tumor entities, and it is strongly increased in a subgroup of meningioma.

Overall, the different tumor types are characterized by different amount of intensity changes. This creates a sort of compositional “fingerprint” that can be exploited not only to discern between non-tumor and tumor tissue, but also to distinguish among tumor types. The differences of intensity of Raman bands give insights in the biochemical alterations related to brain neoplasia. However, they are not directly suited for diagnostic purposes, as the overlap of ranges is too large to enable discerning tissue type. For sound detection of tumor and a tumor diagnosis, a supervised classification approach is required.

### Classification of Neoplastic vs. Non-neoplastic Brain Tissue

Prior to classification, dimensionality reduction was obtained by principal component analysis (PCA), which performs a linear decomposition of signals in spectral components (vectors) and scores. The same approach was used for fluorescence and Raman signals.

The vectors of PCA performed on fluorescence signal exhibit different bands, whose attribution to biochemical compounds is not possible. However, the scores up to principal component (PC) n. 16 display significant differences between non-tumor and tumor tissue (two-tailed Mann-Whitney test, *P* < 0.05; see Supporting Figure S3) and thus can be used to classify tissue types.

The vectors of the PCA performed on Raman signals display bands that can be attributed to biochemical compounds and interpreted based on the above described analysis of spectra. A statistical analysis of the scores shows that several of them up to PC n. 16 are significantly different between tissue classes (two-tailed Mann-Whitney test, *P* < 0.05; see Supporting Figure S4). They mainly account for the different content of lipids and proteins as well as calcifications and carotenoids.

The result of classification based on quadratic discriminant analysis of PCA scores is given in Table 3. The classification of fluorescence data enabled to correctly classify more than 90% of spectra and about 95% of biopsies. However, correct rates for astrocytoma and oligodendroglioma were notably lower. One non-neoplastic tissue biopsy was classified as tumor. By performing the classification using Raman data, the correct rate of spectra classification is slightly better, while the correct rate for classification of biopsies is similar. Also in this case, the ability to recognize astrocytoma and oligodendroglioma is worse compared to other types of tumors, but, importantly, all non-neoplastic biopsies were correctly classified.

**Table 3:** Classification of neoplastic vs. non-neoplastic brain tissue.

			**Fluorescence**				**Raman**				**Combined**			
**Biopsy type**	**No. of spectra**	**No. of biopsies**	**Correct classified spectra**		**Correct classified biopsies**		**Correct classified spectra**		**Correct classified biopsies**		**Correct classified spectra**		**Correct classified biopsies**	
			**No**.	**%**	**No**.	**%**	**No**.	**%**	**No**.	**%**	**No**.	**%**	**No**.	**%**
Non tumor	37	7	30	81	6	86	31	84	7	100	32	86	7	100
Astrocytoma	80	14	60	75	10	72	57	71	10	72	62	78	12	86
Oligodendroglioma	50	10	43	86	8	80	37	74	8	80	40	80	9	90
GBM	261	52	221	85	48	92	235	90	48	92	241	92	49	94
GBM recurrent	105	21	95	90	21	100	103	98	21	100	103	98	21	100
Necrosis	25	4	24	96	4	100	25	100	4	100	25	100	4	100
Metastases	119	23	114	96	23	100	119	100	23	100	119	100	23	100
Meningioma	265	53	260	98	53	100	265	100	53	100	265	100	53	100
Schwannoma	38	8	37	97	8	100	38	100	8	100	38	100	8	100
Others	90	17	81	90	17	100	82	91	17	100	83	92	16	94
Total	1070	209	965	90	198	95	991	93	198	95	1008	94	202	97

**Figure 4:**
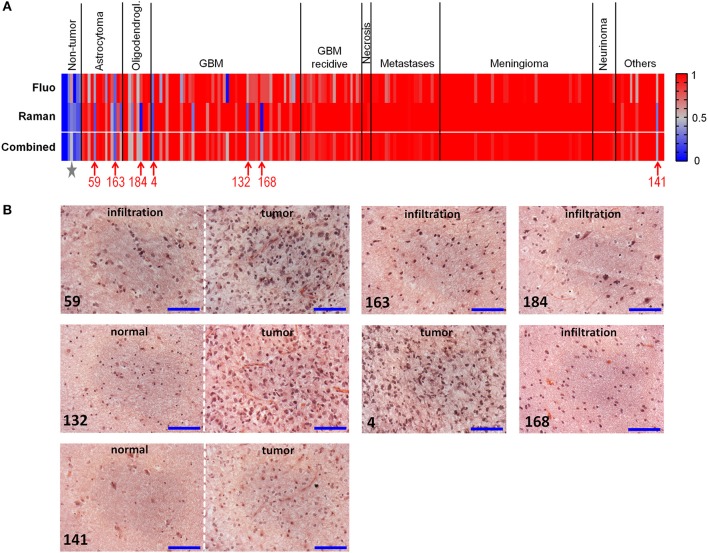
Classification of neoplastic vs. non-neoplastic brain tissue. **(A)** Classification of biopsies obtained from fluorescence and Raman data, and combined classification obtained by performing a mean of probabilities based on fluorescence and Raman data. Probability = 1 corresponds to neoplastic tissue (red). Probability = 0 corresponds to non-neoplastic tissue (blue). Misclassified tumor biopsies are indicated with the patients' number; the star indicates the non-neoplastic biopsy of patient 83, which was misclassified based on fluorescence data. **(B)** Hematoxylin and eosin staining of misclassified biopsies. Patients 59 and 163: diagnosis of astrocytoma WHO grade III; patient 184: diagnosis of oligodendroglioma WHO grade III; patients 4, 132, and 168: diagnosis of GBM; patient 141: diagnosis of dysembryoplastic neuroepithelial tumor WHO grade I. Biopsies of patients 59, 132, 141, 168, 184 contained the infiltration border, including tumor and/or regions of normal tissue; the biopsy of patient four was entirely tumor. Scale bar: 100 μm.

Figure 4A graphically shows the results of classifications by plotting the probabilities of class membership obtained from analysis of fluorescence and Raman data. In both cases, the presence of single misclassified spectra did not prevent the clear attribution of the biopsy, explaining the overall higher correct rate of biopsies compared to spectra. Both approaches fail in recognizing neoplastic from non-neoplastic tissue just in a few cases. Therefore, the use of a combined classification obtained by averaging the classification probabilities obtained from Raman and fluorescence data significantly improved the classification rate, enabling to correctly recognize 97% of tumors (195/202) and 100% of non-neoplastic biopsies (7/7). Also the ability to recognize astrocytoma and oligodendroglioma as tumor was substantially improved by such a combined approach.

Misclassified spectra were further analyzed in order to evaluate the presence of possible artifacts that might have caused the error.

All spectra had regular Raman and fluorescence signals. Therefore, we concluded that no problems occurred during acquisition (e.g., wrong focus setting). Presence of blood contamination was then considered as possible source of misclassification. Spectra containing an evident contribution from blood were identified based on the ratio of intensity of the Raman band at 1,563 cm^−1^ (ν_2_ vibration of hemoglobin) and the intensity of the amide I band at 1,660 cm^−1^, which identifies the tissue (Supporting Figure S5). The Raman signal was considered contaminated with blood if I(1,563 cm^−1^)/I(1,660 cm^−1^) ≥0.4. Overall, 42 spectra were found with a ratio comprised between 0.4 and 2.1, distributed among almost all tissue types (Supporting Table S2). All contaminated spectra were correctly recognized based on fluorescence classification. One contaminated spectrum of normal tissue and one of astrocytoma were assigned to the wrong class based on Raman classification. Therefore, blood contamination is not a cause of misclassification.

Further analysis revealed that all misclassified Raman signals of tumors display in fact spectral features that are typical of normal tissue, with unusual high content of lipids. On the other side, all misclassified Raman signals of normal tissue display lower lipid content (compare Supporting Figure S6 and Figure 2). Therefore, the misclassification is reflecting tissue biochemical properties, rather than being driven by technical issues of the measurement.

Histopathological analysis of the misclassified biopsies made by an experienced neuropathologist on H&E stained sections provided possible error explanations (Figure 4B). The misclassified astrocytoma biopsies both contained the infiltrative border, and were partly or entirely composed of normal tissue. Similarly, the misclassified biopsy of oligodedroglioma contained the infiltration border and was composed to about 90% of non-neoplastic tissue. Two biopsies of GBM contained the infiltrative border as well, with 50 and 80% non-neoplastic tissue, respectively. Only one GBM biopsy (patient 4) was entirely composed of tumor tissue. The misclassified biopsy of the group of “others” (dysembryoplastic neuroepithelial tumor WHO I) was mainly composed of normal tissue. The non-neoplastic biopsy incorrectly recognized by fluorescence classification (indicated by a star in Figure 4A; fluorescence classification probability = 0.62, Raman classification probability = 0.32, combined classification probability = 0.47) originated from the epilepsy surgery of the patient where histopathology revealed abnormal structure (patient 83). These findings provide an explanation of the spectral characteristics mentioned above for misclassified spectra.

### Classification of Tumor Types

**Figure 5:**
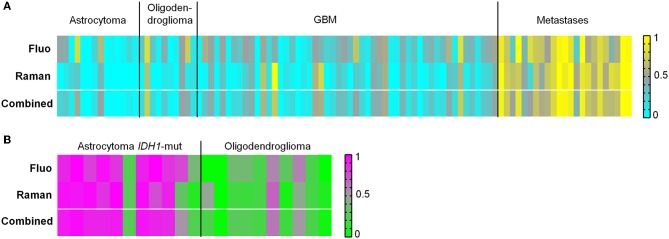
Classification of tumor types. **(A)** Classification of biopsies of glioma vs. biopsies of brain metastases obtained from fluorescence and Raman data, and combined classification obtained by performing a mean of probabilities based on fluorescence and Raman data. Probability = 1 corresponds to metastases (yellow). Probability = 0 corresponds to glioma (cyan). **(B)** Classification of biopsies of astrocytoma *IDH1*-mut vs. biopsies of oligodendroglioma obtained from fluorescence and Raman data, and combined classification obtained by performing a mean of probabilities based on fluorescence and Raman data. Probability = 1 corresponds to astrocytoma (pink). Probability = 0 corresponds to oligodendroglioma (green).

The ability to discern glioma and metastases was tested as well. The results are illustrated in Figure 5A and reported in Supporting Table S3. Classification of glioma vs. metastatic biopsies with an overall correct rate above 80% was obtained based on fluorescence signals. However, many biopsies were classified with a probability close to 0.5. Using Raman data, the classification resulted in overall correct rate above 90% and clear-cut probabilities. In this case, the combination of Raman and fluorescence classifications does not improve the result, not only because the correct rate obtained from fluorescence is lower, but also because the two approaches tend to misclassify the same biopsies.

The type of metastases had no apparent influence on the misclassification in our dataset. The three biopsies misclassified based on Raman data are lung, breast and colon adenoma metastases. No clear correlation between misclassification and presence of blood contamination was found (Supporting Table S2). Histopathological analysis of the misclassified biopsies revealed that three GBM biopsies contained large necrotic areas. In the majority of misclassified GBM biopsies, the tumor tissue was characterized by small cells with thin cytoplasm, somehow similar to lung carcinoma, although with variable degree of similarity depending on the biopsy. The misclassified biopsies of metastases contained the tumor border and were partly composed of non-neoplastic or stromal tissue. Histological images are shown in Supporting Figure S7.

Next, it was evaluated whether astrocytoma with *IDH1*-mutation (*IDH1*-mut) could be identified vs. oligodendroglioma. A classification correct rate of about 80% was obtained for biopsies using either fluorescence or Raman data (Figure 5B and Supporting Table S4). A combination of Raman and fluorescence classification probabilities does not improve the result, because the two approaches tend to misclassify the same biopsies. The histopathological analysis revealed that most misclassified biopsies possessed morphological features typical of the other tumor type, or contained the tumor border with regions of non-neoplastic tissue. Histological images are shown in Supporting Figure S8.

## Discussion

Several studies have already shown that Raman spectroscopy is a very powerful and sensitive technique to highlight subtle aspects of brain tumor biochemistry (12, 17, 18, 22) and to monitor tumor metabolism (9, 31). Our study shows that Raman spectroscopy enables to distinguish neoplastic from non-neoplastic tissue without a pre-existing knowledge about tumor type, by exploiting the overall biochemical differences that characterize neoplastic tissue compared to brain parenchyma. The tumors are generally characterized by decreased lipid content and increased protein content in comparison to non-tumor tissue, in agreement with previous studies (11, 12, 17, 18, 22). These changes were exploited by supervised classification, which allowed distinguishing tumor from non-tumor tissue with high sensitivity and specificity. Incorrect recognition of tumor biopsies was mainly related to presence of low-infiltrative and non-neoplastic regions, as revealed by histopathology. Therefore, it might be that the Raman measurements of those biopsies have been performed on normal tissue regions and the classification results were in fact correct.

Each tumor type is characterized by a specific spectral signature, which enables to recognize different entities. It is possible to distinguish glioma vs. metastases, which can have important effects on surgical approach and therapy. A history of systemic cancer is helpful in differentiating metastatic brain tumor from GBM, but a primary lesion is sometimes not known. The diagnosis of GBM vs. single brain metastasis based on anatomic MR imaging is problematic, because of their similar imaging appearance, and may require a surgical biopsy or specially refined imaging techniques (32). Spectroscopy could deliver intraoperative information with good reliability. Prerequisite for successful application is a measurement on vital tumor tissue. The presence of necrosis or of normal tissue hampers the recognition of tumor type, as shown by histopathological analysis of misclassified biopsies.

Based on the most recent WHO recommendations for brain tumor classification, different entities are meanwhile defined by the combination of histopathological and molecular parameters (33). The results of this study strongly suggests that Raman spectroscopy enables to distinguish *IDH1*-mut astrocytoma and oligodendroglioma—which differ for the presence of 1p/19q codeletion—thus showing potential for diagnostics. However, the number of patients in these groups was too small to retrieve sound results, and our findings need to be confirmed in larger studies.

Combined exploitation of the spectroscopic information contained in both fluorescence and Raman signals was used to discern tumor vs. non-tumor tissue with improved sensitivity and specificity. It is well-known that fluorescence often accounts for the major part of the signal which is acquired during a Raman measurement on biological tissue (34). In Raman spectroscopy, the presence of an autofluorescence background is in fact usually considered a pitfall rather than a source of information. Fluorescence spectra of biological tissue are composed by much broader and overlapping bands compared to Raman spectra, and they are widely regarded as uninformative. In all previous spectroscopic studies, a big effort was dedicated to the elimination of this unwanted signal and retrieve the pure Raman spectrum, most commonly using mathematical data pre-processing (35).

Endogenous cellular fluorophores include nicotinamide adenine dinucleotide (NADH), NADH phosphate (NADPH), flavin adenine dinucleotide (FAD), lipofuscin, retinoids, and extracellular proteins like collagen and elastin. Some of these fluorophores bind to cellular proteins slightly changing the fluorescence spectra. Therefore, the fluorescence signatures provide insights into cellular processes and tissue types (36). Consistently, we found significant differences in intensity among brain tissue and tumor types and used the spectral information for classification. We have already shown that the spectral information of near infrared fluorescence acquired in a Raman measurement can be used to retrieve biological information (37). On the other side, it was shown elsewhere that visible fluorescence can be used for brain tumor detection (38). Our data demonstrate that the fluorescence is especially useful to discern tumor and non-neoplastic tissue. It carries also information about tumor entities, but Raman spectra provide better classification results for tumor type recognition.

The used approach is also stable against experimental issues, such as presence of blood in the tissue. The use of large excitation and collection fibers with a low magnification objective (10× with NA = 0.25) allowed sampling of a large tissue volume due to the large laser spot, in order to compensate for local tissue heterogeneity (e.g., presence of small blood vessels). On the other side, the large depth of focus released the constraint of precise focus setting, making the measurement easier. Therefore, all measurements could be correctly performed by medical personnel after a short training, and no specialists were required for control or supervision. The measurements could be integrated in the surgical routine without any further effort of the operating team and without leading to prolonged surgery duration.

The analysis of resected biopsies as presented here offers important advantages in the perspective of immediate translation to the clinics. It does not carry any additional risk for the patients and can be applied in all cases where a biopsy can be obtained. Given the availability of a Raman system near the OR, our approach is directly suited for large clinical studies, with no impact on the surgical workflow and without the need of a specialist on site. Therefore, our approach can be immediately employed in large multicenter studies that constitute the next step required for assessment of Raman spectroscopy in brain surgery. Such studies will enable to analyze larger numbers of tumors in relatively short time and thus provide a representative amount also of rare diseases, as well as a larger number of non-neoplastic samples. In the frame of multicenter studies, standardization issues may be properly addressed to increase the generalizability of results. Very large datasets will enable also to refine the mathematical algorithms and improve the stability of classification also in case of low infiltrative brain parenchyma.

Intrinsic limitation of an approach based on measurement of biopsies is the impossibility to provide immediate feedback to the surgeon about tissue still *in situ*. Raman spectroscopy of biopsies is rather a new and fast approach for intraoperative histopathology. A second main limitation of the research lies in the scarce availability of non-neoplastic tissue, which consisted of tissue from epileptic patients. Although histopathological analysis of cryosections revealed mostly normal morphology, the spectral characteristics of the measured samples might be not fully representative for healthy brain tissue. As normal functional brain tissue is never excised from patients, the only possible alternative to obtain reference tissue is from autopsies. However, the time elapsed between death and tissue isolation can be up to few days and alterations of tissue micromorphology and biochemistry are thus expected. In fact, forensic studies have highlighted that spectroscopy of brain tissue can be used to measure the *post mortem* interval (39). Tumor patient biopsies taken at the tumor border may occasionally contain non-tumor tissue. The peritumoral region of high grade glioma and brain metastases displays several alterations including inflammation and astroglial activation (40, 41), but may still provide a good reference. Nevertheless, very precise spatial information from pathology is required to exploit these tumor borders for optical measurements, and such a precise histological reference is achievable only when spectroscopy is performed on cryosections. A spectroscopic measurement *in situ* will allow overcoming both limitations, offering immediate feedback to the surgeon and the unique possibility to get reference data from really “normal” brain tissue to confirm the results of studies performed *ex vivo*.

Impressive proof-of-principles already demonstrated that Raman spectroscopy can be also applied *in situ* for intraoperative assessment of glioma and infiltrative zones (26, 42). However, several issues have to be considered for *in situ* measurements. It was shown that standard OR illumination lamps interfere with the Raman measurements, so that dimming or switching off OR illumination (43) and subtraction of the background spectrum, or special adaptation of surgical microscopes (44) are required to obtain reliable spectra of the tissue, although some advanced classification algorithm can also cope with illumination artifacts (45). Further issues are related with the use of a laser source during the measurement, which requires special safety measures for the operating team. Raman probes going in contact with the tissue must be subjected to accurate sterilization procedures before being used on the patients. Their use is currently possible on a small “research” scale, but reports about long-term stability of fibers subjected to repeated cleaning and sterilization procedures are lacking so far. Furthermore, *in situ* Raman analysis is possible only on selected patients, if the tumor is directly visible in the cranial window and the surgeon does not envisage additional risks for the patient. These ethical and practical issues still need to be solved and may hamper in the near future the translation of *in situ* Raman spectroscopy to the clinics.

In conclusion, Raman spectroscopy of brain tumor biopsies is a purely optical technique that allows label-free analysis of brain tumor tissue and that is mature for clinical trials. Here, we showed that Raman spectroscopy of intraoperative biopsies can be performed within few minutes during the surgical routine, and that it allows the identification of neoplastic tissue with high accuracy and sensitivity, beyond the inter- and intra-patient variability. The discrimination of neoplastic vs. normal tissue does not require a pre-existing knowledge about tumor type and diagnostically relevant information can be extracted as well. Measurement protocols are fast and simple: an immediate translation of Raman spectroscopic analysis of biopsies in the surgical practice is possible. These results open new scenarios for intraoperative label-free histopathology, and we envisage that Raman spectroscopy will definitely qualify as adjunct tool for neurosurgery in the near future. Furthermore, the results constitute a strong motivation to develop safe and easy-to-use Raman systems for medical use.

## Data Availability Statement

The raw data supporting the conclusions of this manuscript will be made available by the authors, without undue reservation, to any qualified researcher.

## Ethics Statement

The studies involving human participants were reviewed and approved by Ethics committee at the Dresden University Hospital (EK 323122008). The patients/participants provided their written informed consent to participate in this study.

## Author Contributions

MK and GSt contributed conceptualization of the study. OU and RG developed the experimental design and implementation and wrote the first draft of the manuscript. OU, RG, and MM analyzed and interpreted the data. GSc and EK contributed research facilities and resources. All authors contributed to manuscript revision, read and approved the submitted version.

### Conflict of Interest

The authors declare that the research was conducted in the absence of any commercial or financial relationships that could be construed as a potential conflict of interest.
