# Supplementary material for: Rapid Label-Free Analysis of Brain Tumor Biopsies by Near Infrared Raman and Fluorescence Spectroscopy—A Study of 209 Patients
Source: Front Oncol. 2019 Nov 5;9:1165. doi: 10.3389/fonc.2019.01165 (PMC6848276; doi:10.3389/fonc.2019.01165)
Supplement: Supplementary file 1 [file Data_Sheet_1.PDF]

Supplementary Material

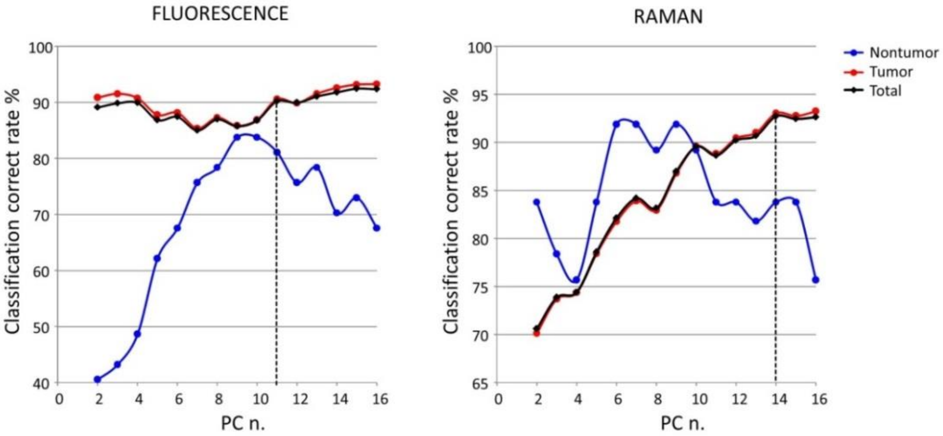

Supporting Figure S1. Discerning neoplastic from non-neoplastic brain tissue: classification correct rate vs. number of PC scores used for classification. The plots of number of scores for non-tumor and tumor tissue, as well the overall correct rate, are plotted against the number of scores used for quadratic discriminant analysis. The best classification was obtained using the first 11 and 14 PC scores for fluorescence and Raman, respectively. It corresponds to the highest overall classification correct rate with non-tumor tissue classification correct rate > 80%.

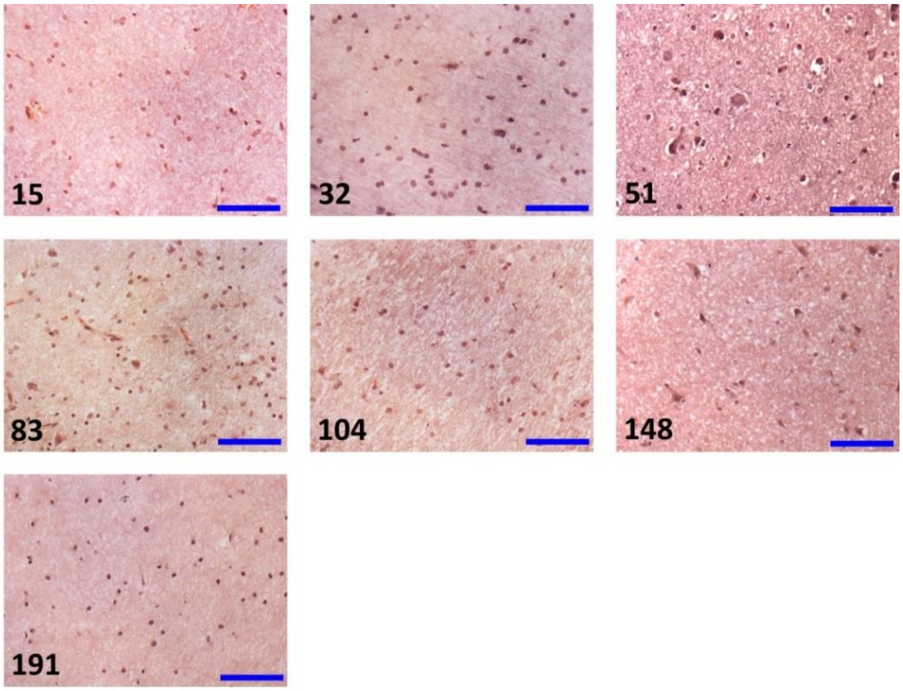

Supporting Figure S2. Hematoxylin and eosin staining of non-neoplastic biopsies.

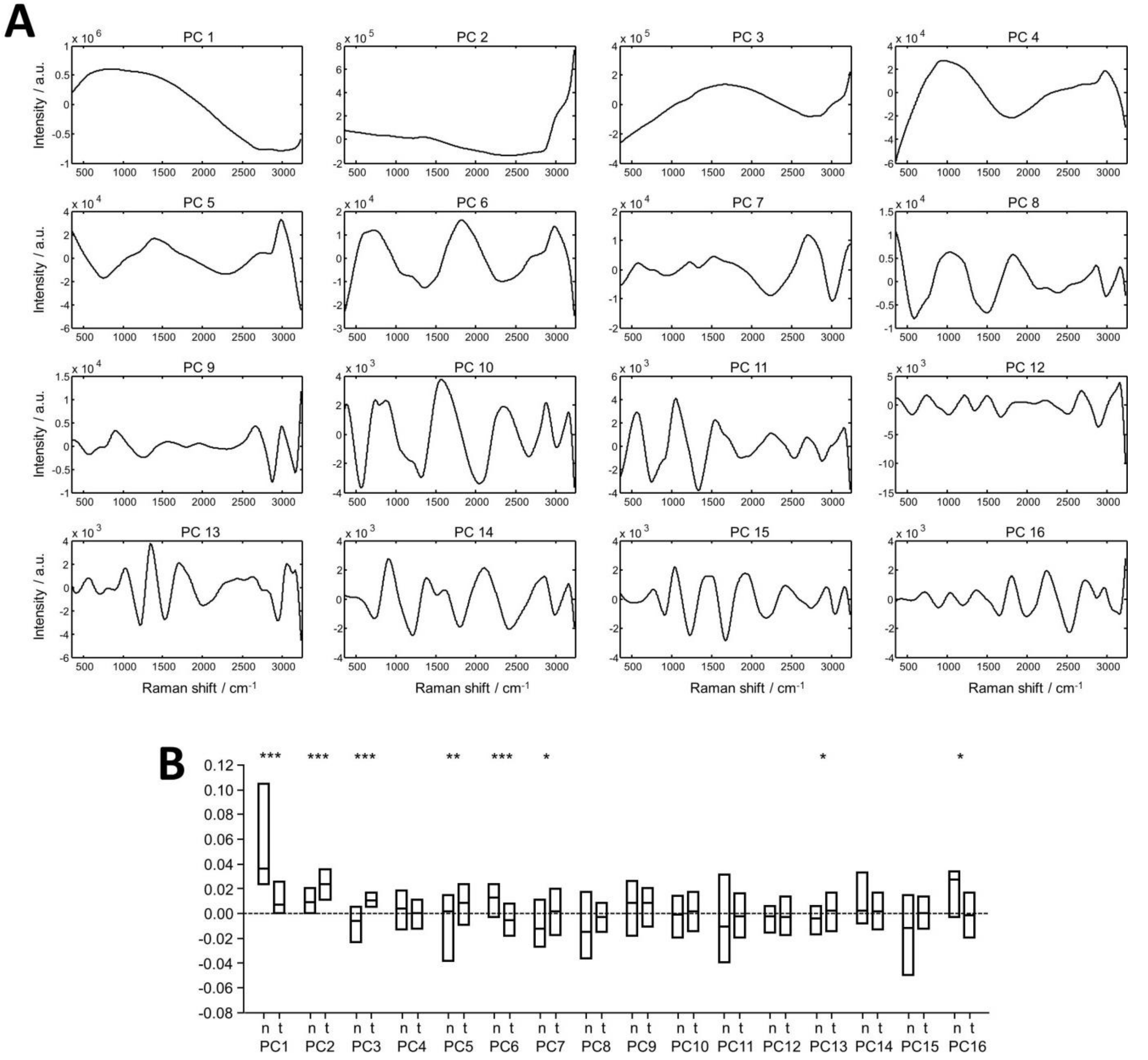

Supporting Figure S3. PCA on fluorescence spectra. (A) vectors. (B) scores for non-tumor (n) and tumor (t) tissue; median, box: 25th-75th percentiles. Two-tailed Mann-Whitney test (*** $P < 0.001$, ** $P < 0.01$, * $P < 0.05$). PC 1 describes a mean fluorescence, and the corresponding scores are higher for non-neoplastic tissue, in accordance with the higher fluorescence intensity shown in Fig. 1.

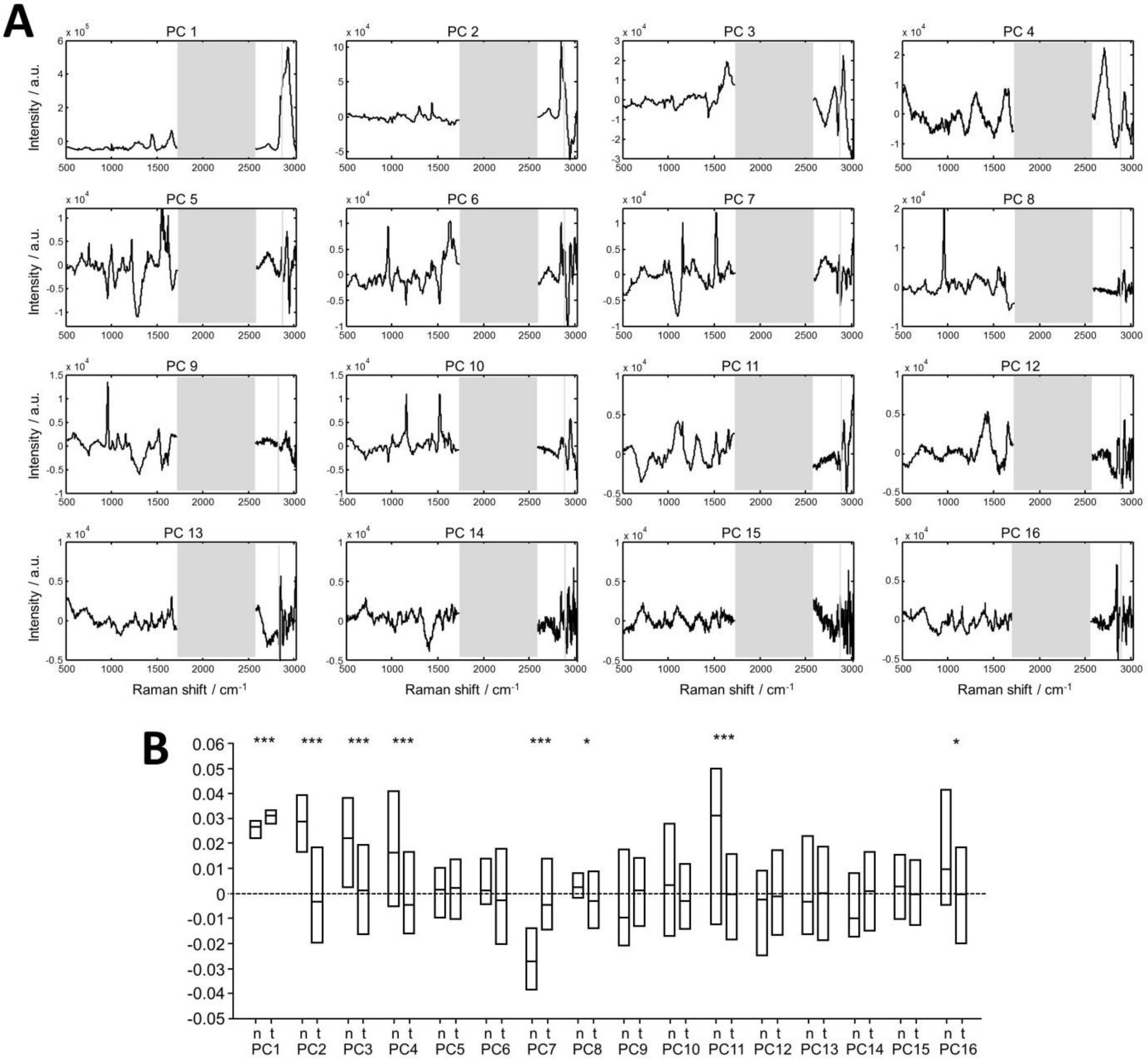

Supporting Figure S4. PCA on Raman spectra. (A) Vectors. (B) Scores for non-tumor (n) and tumor (t) tissue; median, box: 25th-75th percentiles. Two-tailed Mann-Whitney test (*** $P < 0.001$, ** $P < 0.01$, * $P < 0.05$). PC 2 displays all bands of lipids. As expected, the corresponding score is significantly higher for non-neoplastic tissue. PC 3 has intense bands at 1660 and 2950 cm^{-1}, which account for protein. The corresponding score is in fact higher for tumors. PC 7 is dominated by bands of carotene and its score is higher for tumors. PC 8 and 9 mainly account for calcifications. As seen previously, this is an exclusive feature of meningioma, and this justifies the fact that overall the difference between all tumors and non-neoplastic tissue is not always significant. PC 11 and PC 16 likely describe differences in protein content. Interestingly, PC 5 displays bands typical of hemoglobin and represents blood contamination. The score is not different between tissue classes.

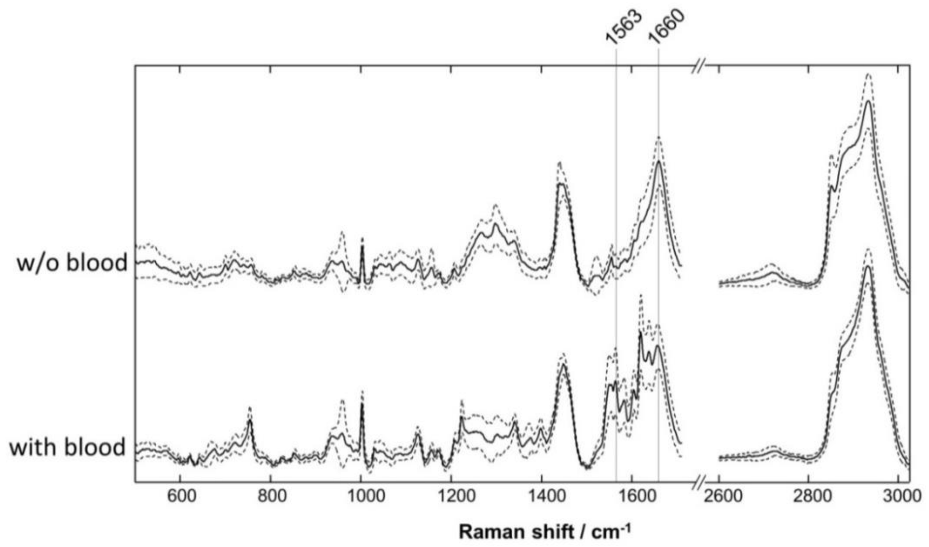

Supporting Figure S5. Contamination of blood. Raman spectra with blood contamination showing evident spectral features of hemoglobin (with I(1563cm^{-1}) / I(1660cm^{-1}) ≥ 0.4) and without blood contamination (mean ± SD).

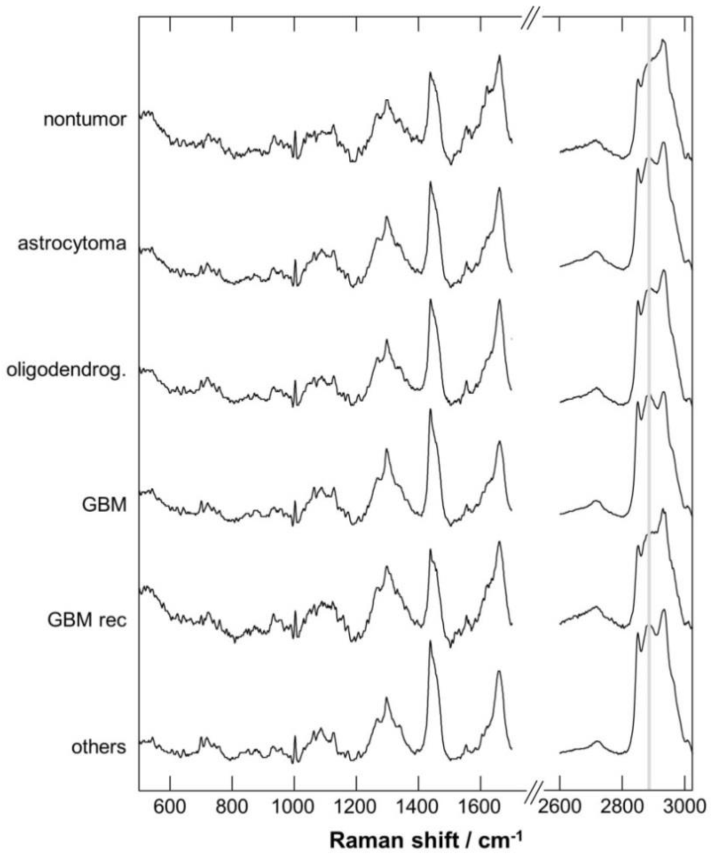

Supporting Figure S6. Discerning neoplastic from non-neoplastic brain tissue: misclassified spectra. The mean spectrum obtained by averaging all misclassified spectra for each tissue type is shown.

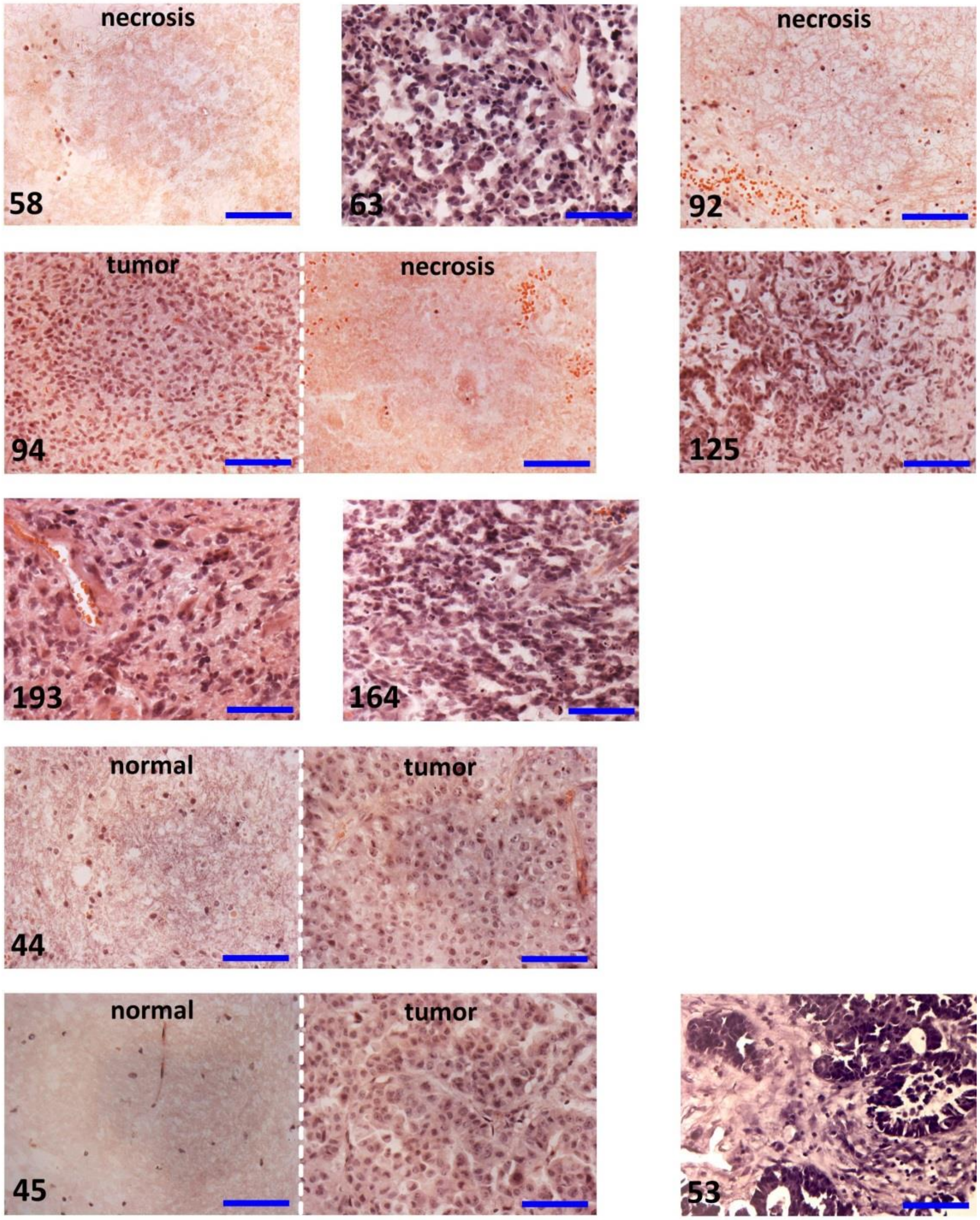

Supporting Figure S7. Hematoxylin and eosin staining of misclassified biopsies of glioma vs. brain metastases (based on Raman data). Patients 58, 63, 92, 94, 125, 193: diagnosis of GBM; patient 164: diagnosis of oligodendroglioma WHO grade III; patients 44, 45, 53: diagnosis of brain metastasis. Scale bar: 100 µm.

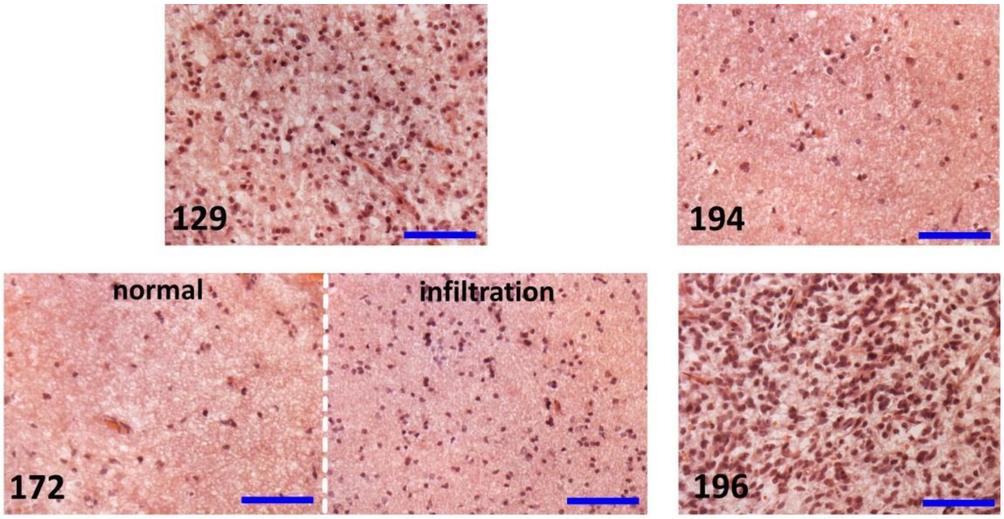

Supporting Figure S8. Hematoxylin and eosin staining of misclassified biopsies of astrocytoma *IDH*-mut vs. oligodendroglioma. Patients 129, 194: diagnosis of astrocytoma WHO grade III *IDH*-mut; the biopsy 129 is a cell-rich tumor with small round cells and few astroglial fibers, thus similar to the morphology of an oligodendroglioma; the biopsy 194 was composed by substantially normal tissue. Patients 172, 196: diagnosis of oligodendroglioma WHO grade III; biopsy 172 contained the infiltration border and normal tissue. Scale bar: 100 µm.

Supporting Table S1. List of patients with diagnosis.

Patient n.	Age	Diagnosis	IDH1 mut	1p/19q cod
**15**	46	non-neoplastic, epilepsy		
**32**	38	non-neoplastic, epilepsy		
**51**	58	non-neoplastic, epilepsy		
**83**	18	non-neoplastic, epilepsy		
**104**	50	non-neoplastic, epilepsy		
**148**	55	non-neoplastic, epilepsy		
**191**	32	non-neoplastic, epilepsy		
**5**	35	astrocytoma, (WHO III)	yes	no
**33**	36	astrocytoma, recurrent (WHO III)	no	partial
**34**	42	astrocytoma, recurrent (WHO III)	yes	no
**38**	37	astrocytoma, recurrent (WHO III)	yes	no
**59**	40	astrocytoma, recurrent (WHO III)	yes	no
**68**	79	astrocytoma, (WHO III)	no	
**102**	74	astrocytoma, (WHO III)	yes	no
**129**	41	astrocytoma, recurrent (WHO III)	yes	no
**134**	31	astrocytoma, recurrent (WHO II)	yes	no
**147**	38	astrocytoma, recurrent (WHO III)	yes	no
**160**	71	astrocytoma, (WHO II)	no	
**163**	49	astrocytoma, (WHO III)	yes	no
**176**	44	astrocytoma, recurrent (WHO III)	yes	no
**194**	49	astrocytoma, recurrent (WHO III)	yes	no
**88**	61	oligodendroglioma, recurrent (WHO III)	yes	yes
**152**	55	oligodendroglioma, (WHO III)	yes	yes
**164**	41	oligodendroglioma, recurrent (WHO III)	yes	yes
**165**	38	oligodendroglioma, recurrent (WHO II)	yes	yes
**167**	63	oligodendroglioma, (WHO III)	yes	yes
**169**	44	oligodendroglioma, (WHO III)	yes	yes
**172**	57	oligodendroglioma, recurrent (WHO III)	yes	yes
**184**	41	oligodendroglioma, recurrent (WHO III)	yes	yes
**196**	52	oligodendroglioma, recurrent (WHO III)	yes	yes
**209**	37	oligodendroglioma, recurrent (WHO III)	yes	yes
**4**	75	glioblastoma, (WHO IV)	no	
**8**	66	glioblastoma, (WHO IV)		
**10**	48	glioblastoma, (WHO IV)	no	
**18**	47	glioblastoma, (WHO IV)	no	
**23**	71	glioblastoma, (WHO IV)	no	
**24**	71	glioblastoma, (WHO IV)	no	
**27**	72	glioblastoma, (WHO IV)	no	
**39**	82	glioblastoma, (WHO IV)	no	
**42**	65	glioblastoma, (WHO IV)	no	
**48**	62	glioblastoma, (WHO IV)	no	
**55**	71	glioblastoma, (WHO IV)		
**58**	84	glioblastoma, (WHO IV)	no	
**62**	75	glioblastoma, (WHO IV)	no	
**63**	85	glioblastoma, (WHO IV)	no	
**66**	52	glioblastoma, (WHO IV)	no	
**72**	28	glioblastoma, (WHO IV)	yes	
**75**	56	glioblastoma, (WHO IV)	no	
**76**	73	glioblastoma, (WHO IV)	no	
**80**	54	glioblastoma, (WHO IV)	no	
**89**	77	glioblastoma, (WHO IV)		
**92**	53	glioblastoma, (WHO IV)	no	
**94**	69	glioblastoma, (WHO IV)	no	
**99**	63	glioblastoma, (WHO IV)	no	
**101**	44	glioblastoma, (WHO IV)	no	

**106**	43	glioblastoma, (WHO IV)	no	
**107**	73	glioblastoma, (WHO IV)		
**108**	59	glioblastoma, (WHO IV)	no	
**114**	52	glioblastoma, (WHO IV)	no	
**118**	65	glioblastoma, (WHO IV)	no	
**119**	81	glioblastoma, (WHO IV)	partial	
**121**	71	glioblastoma, (WHO IV)	no	
**123**	80	glioblastoma, (WHO IV)		
**125**	73	glioblastoma, (WHO IV)	no	partial
**132**	58	glioblastoma, (WHO IV)	no	
**140**	79	glioblastoma, (WHO IV)		
**146**	67	glioblastoma, (WHO IV)		
**151**	81	glioblastoma, (WHO IV)		
**162**	69	glioblastoma, (WHO IV)	no	
**168**	58	glioblastoma, (WHO IV)	no	
**171**	76	glioblastoma, (WHO IV)		
**173**	73	glioblastoma, (WHO IV)	no	
**178**	80	glioblastoma, (WHO IV)	no	
**182**	82	glioblastoma, (WHO IV)		
**187**	67	glioblastoma, (WHO IV)	no	
**189**	75	glioblastoma, (WHO IV)		
**193**	49	glioblastoma, (WHO IV)	no	
**195**	61	glioblastoma, (WHO IV)	no	
**198**	52	glioblastoma, (WHO IV)	no	
**201**	64	glioblastoma, (WHO IV)		
**203**	60	glioblastoma, (WHO IV)	no	
**205**	78	glioblastoma, (WHO IV)	no	
**206**	83	glioblastoma, (WHO IV)	no	
**19**	60	glioblastoma, recurrent (WHO IV), **necrotic tissue**		
**29**	65	glioblastoma, recurrent (WHO IV), **necrotic tissue**		
**31**	49	glioblastoma, recurrent (WHO IV)	yes	yes
**71**	75	glioblastoma, recurrent (WHO IV)		
**79**	49	glioblastoma, recurrent (WHO IV)	no	
**81**	54	glioblastoma, recurrent (WHO IV)		
**87**	50	glioblastoma, recurrent (WHO IV)		
**96**	71	glioblastoma, recurrent (WHO IV)		
**111**	44	glioblastoma, recurrent (WHO IV)	no	
**113**	62	glioblastoma, recurrent (WHO IV),		
**117**	71	glioblastoma, recurrent (WHO IV),	no	
**124**	50	glioblastoma, recurrent (WHO IV)		
**133**	50	glioblastoma, recurrent (WHO IV),		
**139**	50	glioblastoma, recurrent (WHO IV)		
**143**	57	glioblastoma, recurrent (WHO IV),	no	
**150**	65	glioblastoma, recurrent (WHO IV),		
**155**	57	glioblastoma, recurrent (WHO IV)		
**158**	53	glioblastoma, recurrent (WHO IV)		
**174**	31	glioblastoma, recurrent (WHO IV)	yes	
**175**	78	glioblastoma, recurrent (WHO IV)	no	
**180**	23	glioblastoma, recurrent (WHO IV)		
**185**	56	glioblastoma, recurrent (WHO IV)		
**207**	53	glioblastoma, recurrent (WHO IV)	no	
**45**	56	metastasis, adenocarcinoma (breast carcinoma)		
**157**	54	metastasis, adenocarcinoma (breast carcinoma)		
**177**	76	metastasis, adenocarcinoma (breast or lung carcinoma)		
**53**	60	metastasis, adenocarcinoma (colon carcinoma)		
**73**	55	metastasis, adenocarcinoma (colon carcinoma)		
**6**	50	metastasis, adenocarcinoma (colon carcinoma)		

**49**	70	metastasis, adenocarcinoma (lung carcinoma)
**57**	79	metastasis, adenocarcinoma (lung carcinoma)
**181**	75	metastasis, adenocarcinoma (lung carcinoma)
**204**	62	metastasis, adenocarcinoma (lung carcinoma)
**122**	49	metastasis, adenocarcinoma (lung carcinoma)
**137**	69	metastasis, adenocarcinoma (lung carcinoma)
**20**	57	metastasis, adenocarcinoma (lung carcinoma)
**30**	72	metastasis, adenocarcinoma (lung carcinoma)
**44**	58	metastasis, adenocarcinoma (lung carcinoma), recurrent
**82**	69	metastasis, adenocarcinoma (pancreas carcinoma)
**90**	60	metastasis, adenocarcinoma (sarcomatoid)
**154**	63	metastasis, adenocarcinoma (stomach carcinoma)
**37**	**57**	metastasis, clear cell carcinoma (renal carcinoma) - **not measured**
**85**	40	metastasis, melanoma, **necrotic tissue**
**109**	55	metastasis, melanoma, recurrent
**200**	57	metastasis, squamous cell carcinoma (hypopharyngeal cancer)
**97**	81	metastasis, squamous cell carcinoma (lung carcinoma)
**21**	66	metastasis, squamous cell carcinoma (lung carcinoma)
**91**	66	metastasis, squamous cell carcinoma (lung carcinoma), recurrent
**153**	64	metastasis, prostate carcinoma, **necrotic tissue**
**2**	77	meningioma, atypical (WHO II)
**9**	76	meningioma, atypical, recurrent (WHO II)
**11**	68	meningioma, atypical, recurrent (WHO II)
**28**	83	meningioma, atypical (WHO II)
**43**	67	meningioma, atypical, recurrent (WHO II)
**54**	81	meningioma, atypical (WHO II)
**67**	69	meningioma, atypical (WHO II)
**112**	79	meningioma, atypical (WHO II)
**126**	57	meningioma, atypical (WHO II)
**144**	70	meningioma, atypical (WHO II)
**149**	79	meningioma, atypical, recurrent (WHO II)
**170**	14	meningioma, atypical (WHO II)
**179**	80	meningioma, atypical, recurrent (WHO II)
**183**	84	meningioma, atypical (WHO II)
**188**	56	meningioma, atypical, recurrent (WHO II)
**202**	65	meningioma, atypical (WHO II)
**12**	43	meningioma, fibrous (WHO I)
**41**	46	meningioma, fibrous (WHO I)
**105**	54	meningioma, fibrous(WHO I)
**156**	78	meningioma, fibrous (WHO I)
**159**	67	meningioma, fibrous (WHO I)
**192**	58	meningioma, fibrous (WHO I)
**197**	45	meningioma, fibrous (WHO I)
**199**	79	meningioma, fibrous, (WHO I)
**1**	26	meningioma, meningothelial (WHO I)
**25**	49	meningioma, meningothelial (WHO I)
**26**	24	meningioma, meningothelial (WHO I)
**60**	54	meningioma, meningothelial (WHO I)
**61**	64	meningioma, meningothelial (WHO I)
**64**	32	meningioma, meningothelial (WHO I)
**65**	76	meningioma, meningothelial (WHO I)
**74**	79	meningioma, meningothelial (WHO I)
**115**	72	meningioma, meningothelial (WHO I)
**127**	54	meningioma, meningothelial (WHO I)
**130**	56	meningioma, meningothelial, recurrent (WHO I)
**131**	61	meningioma, meningothelial (WHO I)
**135**	82	meningioma, meningothelial (WHO I)
**166**	52	meningioma, meningothelial (WHO I)

**186**	73	meningioma, meningothelial (WHO I)
**190**	61	meningioma, meningothelial (WHO I)
**84**	61	meningioma, microcystic (WHO I)
**100**	43	meningioma, microcystic (WHO I)
**13**	51	meningioma, transitional (WHO I)
**16**	59	meningioma, transitional (WHO I)
**35**	54	meningioma, transitional (WHO I)
**36**	71	meningioma,transitional (WHO I)
**77**	64	meningioma, transitional (WHO I)
**95**	66	meningioma, transitional (WHO I)
**116**	56	meningioma, transitional, recurrent (WHO I)
**138**	52	meningioma, transitional (WHO I)
**145**	37	meningioma, transitional (WHO I)
**161**	51	meningioma, transitional (WHO I)
**211**	43	meningioma, transitional (WHO I)
**3**	75	neurinoma, acustic (WHO I)
**46**	53	neurinoma, conus/cauda (WHO I)
**56**	53	neurinoma, spinal (WHO I)
**69**	41	neurinoma, acustic (WHO I)
**86**	65	neurinoma, acustic, recurrent (WHO I)
**103**	29	neurinoma, acustic (WHO I)
**110**	44	neurinoma, acustic (WHO I)
**142**	65	neurinoma, spinal (WHO I)
**7**	30	other, neurocytoma, extraventricular (WHO II)
**14**	56	other, cavernoma
**17**	55	other, dysembryoplastic neuroepithelial tumor (WHO I)
**22**	76	other, pituitary adenoma, hormone secreting, recurrent
**40**	25	other, ganglioglioma (WHO I)
**47**	62	other, hemangiopericytoma (WHO IIb)
**50**	34	other, ganglioglioma (WHO I)
**52**	42	other, subependymoma (WHO I)
**70**	75	other, pituitary adenoma, partially hormone secreting
**78**	52	other, chordoma, recurrent, (NOS)
**93**	41	other, neurocytoma (WHO II)
**98**	63	other, plexuspapilloma, atypic (WHO II)
**120**	60	other, myeloma (multiple Myeloma of Lambda type)
**136**	19	other, ganglioneuroblastoma (WHO IV)
**141**	29	other, dysembryoplastic neuroepithelial tumor (WHO I)
**208**	49	other, ependymoma, tanycytic (WHO II)
**210**	78	other, squamous cell carcinoma

Supporting Table S2. Discerning neoplastic from non-neoplastic brain tissue: effects of blood contamination. Number of spectra with evident spectral features of hemoglobin (I(1563cm^{-1}) / I(1660cm^{-1}) ≥ 0.4) for each type of biopsy, with relative misclassification rates.

				No. of misclassified spectra with blood contamination					
				Neoplastic vs. non-neoplastic tissue		Glioma vs. metastases		Astrocytoma *IDH*-mut vs. oligodendrog.	
**Biopsy type**	**No. of spectra**	**No. of spectra with blood contamination**	**%**	**Fluorescence**	**Raman**	**Fluorescence**	**Raman**	**Fluorescence**	**Raman**
**Nontumor**	37	1	3	0	1				
**Astrocytoma**	80	3	4	0	1	3	0	1	0
**Oligodendrog.**	50	0	0	0	0	0	0	0	0
**GBM**	261	8	3	0	0	2	2		
**GBM recurrent**	105	3	3	0	0				
**Necrosis**	25	3	12	0	0				
**Metastases**	119	4	3	0	0	0	2		
**Meningioma**	265	15	6	0	0				
**Schwannoma**	38	0	0	0	0				
**Others**	90	6	7	0	0				
**TOTAL**	**1070**	**42**	**4**						

Supporting Table S3. Classification of glioma vs. metastases obtained from fluorescence and Raman data.

			Fluorescence				Raman				Combined			
**Biopsy type**	**No. of spectra**	**No. of biopsies**	**Correct classified spectra**		**Correct classified biopsies**		**Correct classified spectra**		**Correct classified biopsies**		**Correct classified spectra**		**Correct classified biopsies**	
			**No.**	**%**	**No.**	**%**	**No.**	**%**	**No.**	**%**	**No.**	**%**	**No.**	**%**
**Astrocytoma**	80	14	63	84	12	86	76	95	14	100	76	95	12	86
**Oligodendroglioma**	50	10	34	68	7	68	43	86	9	90	41	82	9	90
**GBM**	261	52	193	74	42	81	212	81	46	88	210	80	46	88
**Metastases**	119	23	93	78	19	83	95	80	20	86	93	78	20	86
**TOTAL**	**510**	**99**	**383**	**75**	**80**	**80**	**426**	**84**	**89**	**90**	**420**	**82**	**87**	**88**

Supporting Table S4. Classification of astrocytoma *IDH1*-mut vs. oligodendroglioma obtained from fluorescence and Raman data.

			Fluorescence				Raman				Combined			
**Biopsy type**	**No. of spectra**	**No. of biopsies**	**Correct classified spectra**		**Correct classified biopsies**		**Correctly classified spectra**		**Correct classified biopsies**		**Correct classified spectra**		**Correct classified biopsies**	
			**No.**	**%**	**No.**	**%**	**No.**	**%**	**No.**	**%**	**No.**	**%**	**No.**	**%**
**Astrocytoma**	65	11	53	82	9	82	52	80	8	73	53	82	9	82
**Oligodendroglioma**	50	10	40	80	8	80	40	80	9	90	40	80	8	81
**TOTAL**	**115**	**21**	**93**	**81**	**17**	**81**	**92**	**80**	**17**	**81**	**93**	**81**	**17**	**81**
